# Parafoveal vessel loss and correlation between peripapillary vessel density and cognitive performance in amnestic mild cognitive impairment and early Alzheimer’s Disease on optical coherence tomography angiography

**DOI:** 10.1371/journal.pone.0214685

**Published:** 2019-04-02

**Authors:** Yi Stephanie Zhang, Nina Zhou, Brianna Marie Knoll, Sahej Samra, Mallory R. Ward, Sandra Weintraub, Amani A. Fawzi

**Affiliations:** 1 Department of Ophthalmology, Feinberg School of Medicine, Northwestern University, Chicago, IL, United States of America; 2 Mesulam Center for Cognitive Neurology and Alzheimer’s Disease Center, Feinberg School of Medicine, Northwestern University, Chicago, IL, United States of America; 3 Department of Psychiatry and Behavioral Sciences, Feinberg School of Medicine, Northwestern University, Chicago, IL, United States of America; Massachusetts Eye & Ear Infirmary, Harvard Medical School, UNITED STATES

## Abstract

**Purpose:**

Patients with Alzheimer’s Disease (AD) exhibit decreased retinal blood flow and vessel density (VD). However, it is not known whether these changes are also present in individuals with early AD (eAD) or amnestic type mild cognitive impairment (aMCI), an enriched pre-AD population with a higher risk for progressing to dementia. We performed a prospective case-control clinical study to investigate whether optical coherence tomography angiography (OCTA) parameters in the macula and disc are altered in those with aMCI and eAD.

**Methods:**

This is a single center study of 32 participants. Individuals with aMCI/eAD (n = 16) were 1:1 matched to cognitively normal controls (n = 16). We evaluated OCTA images of the parafoveal superficial capillary plexus (SCP) and two vascular layers in the peripapillary region, the radial peripapillary capillary (RPC) and superficial vascular complex (SVC). Outcome vascular and structural parameters included VD, vessel length density (VLD), adjusted flow index (AFI) and structural retinal nerve fiber layer (RNFL) thickness. We compared these parameters between the two groups and examined the correlation between OCTA parameters and cognitive performance on the Montreal Cognitive Assessment (MoCA).

**Results:**

Cognitively impaired participants demonstrated statistically significant decrease in parafoveal SCP VD and AFI as compared to controls, but no statistically significant difference in peripapillary parameters. Furthermore, we found a significant positive correlation between MoCA scores for the entire study cohort and both the parafoveal SCP VD and peripapillary RPC VLD.

**Conclusion:**

OCTA shows significant decline in parafoveal flow and VD in individuals with early cognitive impairment related to AD, suggesting that these parameters could have potential utility as early disease biomarkers. In contrast, the presence of larger vascular channels in the peripapillary region may have obscured subtle capillary changes in that region. Overall, the correlation between vascular OCTA parameters and cognitive performance supports further OCTA studies in this population.

## Introduction

Effective therapeutic intervention for Alzheimer’s disease (AD) depends on recognizing the disease before the onset of severe clinical symptoms [1, 2]. Prior to the onset of AD, individuals may present with either preclinical AD, defined by the absence of clinical symptoms but positive disease biomarkers such as amyloid and tau proteins in the cerebral spinal fluid (CSF), [3] or they may present with mild cognitive impairment (MCI), defined by mild impairment on neurocognitive testing that does not affect activities of daily living [4]. Individuals with a clinical diagnosis of the amnestic type of MCI (aMCI) represent a high-risk target population that carries a significantly elevated risk, up to 48.7% within 30 months of diagnosis [5], of progressing to AD when compared to those with non-amnestic type MCI [5] or with preclinical AD [6]. Currently, the diagnosis of aMCI relies purely on clinical evaluation, including neuropsychological testing [4]. However, the presence of AD biomarkers in the form of amyloid or tau proteins in the CSF and positron emission tomography with amyloid ligand may help predict progression from MCI to AD [4]. Unfortunately, obtaining these biomarkers remains invasive, costly, and not definitive for clinical practice [7, 8]. Alternative biomarkers maybe be present in the eye as the retina is an extension of the central nervous system developmentally [9]. Thus, cerebral pathology affecting AD may be reflected in the eye and detected with non-invasive optical imaging tools [10].

Optical coherence tomography angiography (OCTA) is a non-invasive clinical tool that can capture the retinal capillary microcirculation at the micrometer resolution [11]. Previous retinal vascular studies using retinal functional imager [12] and laser flowmetry [13] have shown decreased flow in the temporal retinal vein and major parafoveal arterioles and venules in AD and MCI individuals. However, OCTA provides a unique opportunity to investigate the microvasculature in a specific retinal vascular plexus of interest [14, 15]. OCTA has demonstrated that retinal neural sub-layers are supplied by distinct capillary plexuses, each reflecting the metabolic demand of a particular neuronal layer [15]. Importantly, we know that the inner retina layer in both the macula and optic disc bears the brunt of AD pathology including the loss of ganglion cells [16, 17], thinning of the retinal nerve fiber layer (RNFL) [18], and deposition of Aβ plaques [19] according to histological and OCT structural imaging studies.

Two previous OCTA investigations in AD have identified decreased macular vessel density and flow in participants with a wide range of severity of AD (Mini-Mental State Examination average score <20) as compared to cognitively normal controls [20, 21]. One of these studies included MCI participants but did not use age-matched controls or find inner retinal changes [20]. Most recently, O’Bryhim et al. [22] found that those with preclinical AD showed larger foveal avascular zones (FAZ) on OCTA, suggesting vessel drop in the macula. However, none of these studies have specifically focused on aMCI participants, who are a critical population to study due to their high risk of disease progression [5]. Furthermore, OCTA vascular changes surrounding the optic disc remain understudied. Our study addresses these gaps in scientific knowledge by investigating OCTA changes in both the macula and the optic disc in a well-defined cohort of early Alzheimer’s type dementia (eAD) and aMCI participants as compared to matched controls.

## Methods

### Study population

This is a single institution case-control study of early cognitively impaired individuals with aMCI or eAD and cognitively normal participants in the Department of Ophthalmology, Northwestern University in Chicago, Illinois. We recruited participants who had been given a clinical diagnosis of aMCI [4] or eAD [23] as well as matched cognitively normal controls from the Mesulam Center for Cognitive Neurology and Alzheimer’s Disease (CNADC) at Northwestern as described below. We combined eAD and aMCI participants because of their shared presumed underlying AD biology [4] and their similar severity (mild). The study was approved prospectively by the Institutional Review Board of Northwestern University and conducted in accordance with the Health Insurance Portability and Accountability Act of 1996. Written informed consent was obtained from all participants before enrollment. Capacity to provide informed consent was assessed using the University of California, San Diego Brief Assessment of Capacity to Consent (UBACC) [24]. In addition, all participants had a cognitive score of ≥ 13 on the Montreal Cognitive Assessment, the equivalent of ≥19 on the Mini-Mental State Exam (MMSE) [25], which is highly associated with intact decisional capacity [26].

Exclusion criteria for all groups included pre-existing ocular pathology such as glaucoma (history or intra-ocular pressure (IOP) >21), macular degeneration, diabetic or hypertensive retinopathy, retinal detachment, ocular trauma, high myopia (>6D), or extensive cataracts confirmed both by participant report, electronic medical record, and review of OCT images for pathology. Participants with neurological disorders known to affect retinal pathology such as multiple sclerosis and Parkinson’s disease, as well as medical conditions such as uncontrolled hypertension or diabetes, and current smokers were also excluded.

We only included eyes that produced OCTA images without the presence of confounding pathology such as epiretinal membranes or retinal detachment in our analysis. All eyes included also had a signal quality measure (SQ) of ≥6, an absolute signal strength index (SSI) of ≥50 for the macula and SSI ≥45 for the disc, and no significant movement or shadow artifacts. The SQ is an integrated score of SSI as well as motion and shadow artifacts.

### Clinical assessment

All participants underwent extensive annual cognitive assessment at the CNADC using the Uniform Data Set (UDS) of the National Institute on Aging Alzheimer’s Disease Program [27]. The UDS version 3 includes information on demographics, medical history, medication history, as well as clinical, functional, and neuropsychological assessments [28]. Neuropsychological tests conducted include the Montreal Cognitive Assessment (MoCA), Craft Story 21 immediate and delayed recall, and tests of language, visuospatial functions, executive attention, and working memory. Since memory impairment is the primary cognitive deficit in the aMCI and eAD cohort [23], we focused on memory-related tests in the present study. Craft Story immediate and delayed recall tests examine episodic memory while the delayed recall portion of the Rey Auditory Verbal Learning Test (RAVLT) focuses on word list memory. We also included the MoCA score, which assesses overall cognitive function, and is widely used in cognitive aging and dementia research. The MoCA also measures word list retention, category fluency, and orientation, which account for 12 of the 30 points on this test. These are all key symptoms of cognitive impairment in early AD [29]. On all measures used in this study, a higher score indicated better performance. We obtained the most recent cognitive test scores and clinical diagnosis within a year of the experimental testing. The interval between cognitive testing and experimental imaging is reported in Table 1.

**Table 1:** Demographics information.

	aMCI (n = 13)/eAD (n = 3)	Control (n = 16)	P value^a^
**Gender** (male/female)	3/13	3/13	
**Age** (years)	73.03 ± 8.24	73.60 ± 7.69	0.80
**Education** (years)	16.50 ± 2.13	16.56 ± 1.75	0.93
**Race** (n)			
African American	1	1	
Hispanic	1	0	
Caucasian	14	15	
**Diabetes** (n)^b^	2	0	
**Hypertension** (n) ^b^	6	5	
**IOP** (mmHg)	16.19 ± 2.46	16.38 ± 2.22	0.82
**Time Lapse Between Cognitive and Imaging Visits** (years)	0.49 ± 0.45	0.40 ± 0.44	0.57
**MoCA** (total = 30)	20.25 ± 3.80	27.06 ± 2.21	**<0.001**^a^
**CDR**^c^	0.56 ± 0.17	0 ± 0	**<0.001**^a^
**CS Imm** (total = 25)	9.56 ± 2.70	17.00 ± 2.68	**<0.001**^a^
**CS Del** (total = 25)	5.38 ± 4.53	16.19 ± 2.88	**<0.001**^a^
**RAVLT Del** (total = 15)	2.06 ± 2.79	10.81 ± 3.17	**<0.001**^a^

Data reported as mean ± SD with p values from Student’s T-Test or Mann-Whitney U test.

^a^Statistically significant p value at P<0.05

^b^Participants with uncontrolled diabetes or hypertension or with retinopathy related to medical disease were excluded

^c^CDR scores range from 0 (no dementia) to 0.5 (questionable), 1.0 (mild), 2.0 (moderate), 3.0 (severe) dementia

Abbreviations: IOP = intraocular pressure; MoCA = Montreal Cognitive Assessment, CDR = Clinical Dementia Rating; CS Imm = Craft Story Immediate; CS Del = Craft Story Delayed; RAVLT Del = Rey Auditory Verbal Learning Test

The diagnosis of eAD or MCI was based on established research diagnostic criteria [4, 23], with aMCI identified in MCI participants with a predominant memory domain impairment. Whenever possible, available biomarker data were also included in consideration of the final diagnosis. In our study, eAD and MCI were combined together as they are on the early spectrum of cognitive impairment, with MoCA ≥ 13 (equivalent MMSE ≥ 19 [25]). eAD and MCI participants score similarly on the Clinical Dementia Rating (CDR), [27] which is a cognitive impairment metric with 0.5 typically assigned to individuals with MCI and 0.5–1.0 (mild dementia) to those with eAD [30]. However, the diagnosis of eAD also requires mild impairments in activities of daily living while MCI requires perseverance of activities of daily living. Therefore, each participant in the CNADC had a study partner to complete the Activities of Daily Living Questionnaire, a reliable assessment of functional ability in dementia [31, 32]. All cognitively normal controls were 1:1 case-control matched based on age (within three years), gender, and race and underwent the same extensive battery of cognitive research testing.

### Ophthalmological assessment

Each participant underwent manual refraction and best-corrected visual acuity determination of each eye. IOP was assessed using Tonopen (Reichert Technologies, Buffalo, NY, USA). All participants underwent pupil dilation before imaging (installation of proparacaine 0.5%, tropicamide 1%, and phenylephrine 2.5% ophthalmic solutions). Subsequent OCT and OCTA scans were reviewed by a board-certified ophthalmologist for retinal pathology as part of the exclusion criteria.

### Image acquisition and analysis

**Fig 1:**
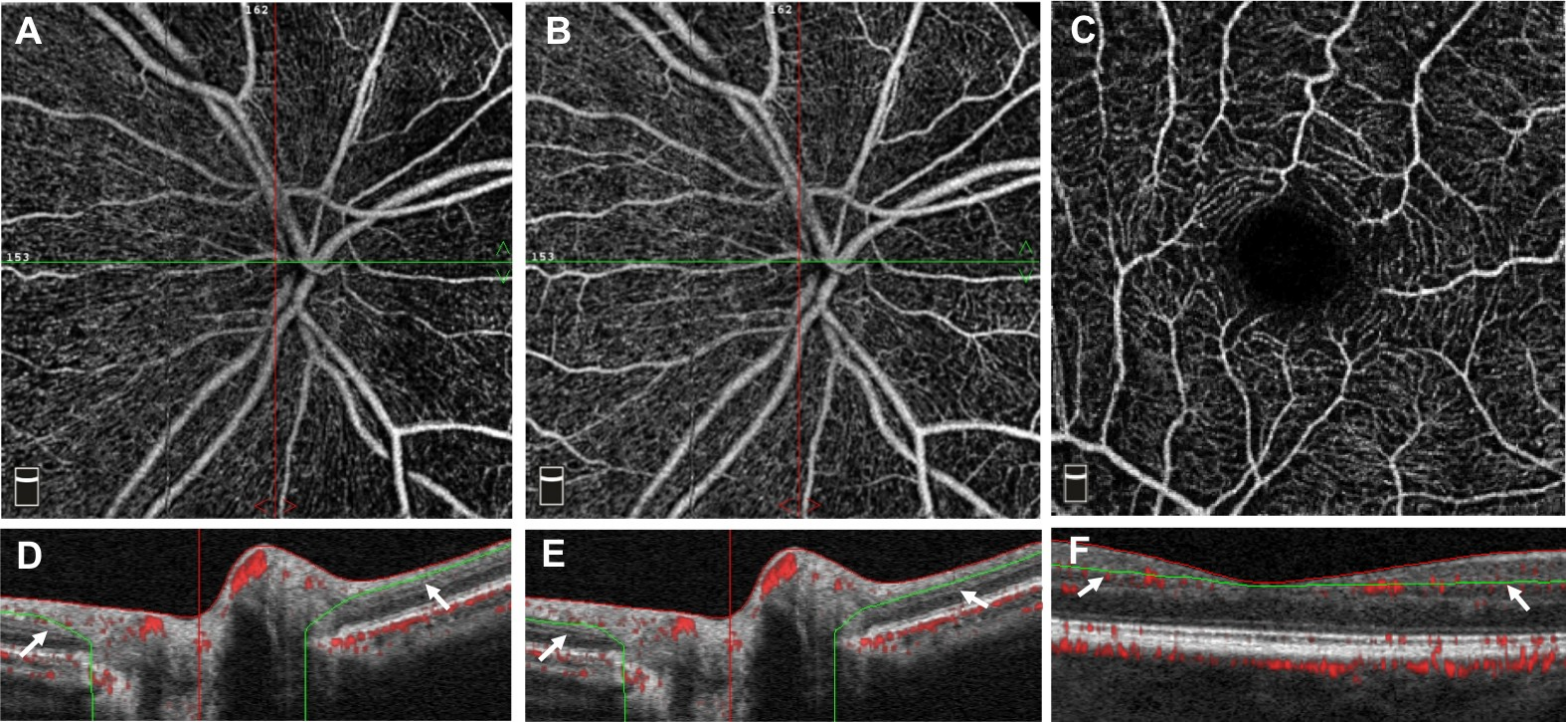
Optical coherence tomography angiography (OCTA) segmentation of the disc and macula. (A) shows a 4.5 x 4.5mm^2^ *en face* OCTA image of the radial peripapillary capillary (RPC) centered on the optic disc of a healthy control, and (D) shows the cross-section segmentation of the RPC. (B) shows the *en face* image of the superficial vascular complex (SVC) segmentation obtained from the same individual with (E) showing the cross-section of SVC. (C) shows 3.0 x 3.0mm^2^ *en face* scan of the superficial capillary plexus (SCP) centered on the of the fovea of the same individual with (F) showing the cross-section segmentation of the SCP.

We used the RTVue-XR OCT Avanti System (Optovue Inc, Fremont, California, USA. Software Version 2016.1.0.26) with split-spectrum amplitude-decorrelation angiography (SSADA) software, described in detail previously [33]. This machine captured 70,000 A-scans per second with a light source centered at 840nm and two consecutive B-scan (M-B frame) each containing 304 A-scans. The SSADA algorithm then extracted blood flow information by quantifying the decorrelation value, which represents differences in signal intensity between consecutive B-scans of the same location on the retina. We obtained 3.0 x 3.0mm^2^ OCT angiograms of the macula, which were automatically segmented to define the superficial capillary plexus (SCP) from the internal limiting membrane (ILM) to the outer boundary of the inner plexiform layer (IPL) (Fig 1C), the deep capillary plexus (DCP) from the inner nuclear layer to the outer boundary of the outer plexiform layer, and the whole retina which contains both the SCP and DCP. For optic nerve imaging, we obtained 4.5 x 4.5 mm^2^ images. Automatic segmentation generated the radial peripapillary capillary (RPC) layer, segmented from the ILM to the outer boundary of the RNFL, and the superficial vascular complex (SVC) layer, from the ILM to the outer boundary of the ganglion cell layer (GCL) (Fig 1A and 1B).

**Fig 2:**
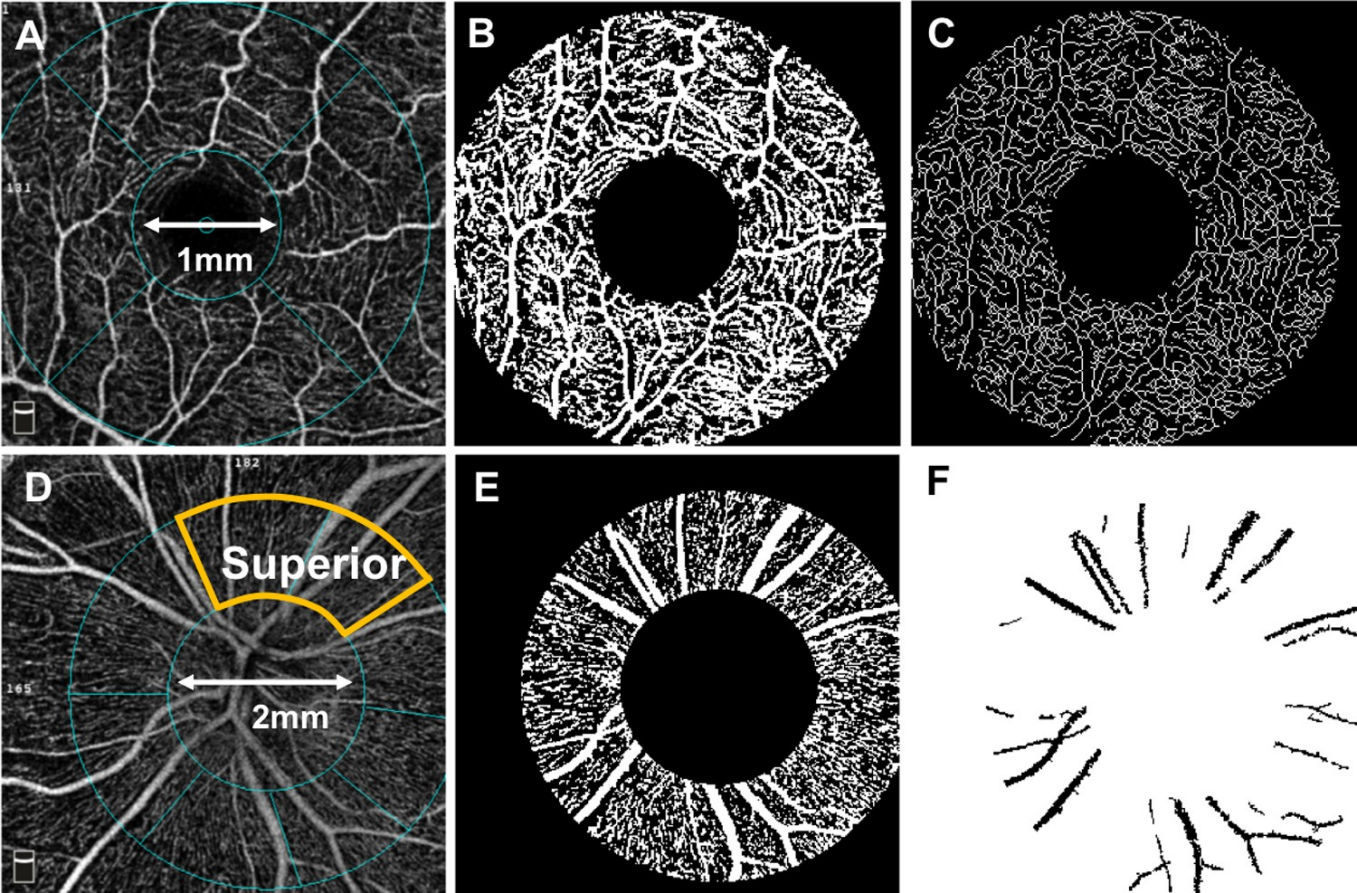
Parafoveal and peripapillary image analysis. A healthy control’s angiograms show the superficial capillary plexus (SCP) of the macula with delineation of the parafovea between rings of 1.0mm and 3.0mm (A), after binarization of parafoveal vessels based on a background defined by the foveal avascular zone (B), and after skeletonizing all vessels to be one pixel wide (C). Bottom row shows the radial papillary capillary (RPC) layer of the same individual with the peripapillary area between rings of 2.0mm and 4.0mm centered on the optic disc and the superior quadrant of the peripapillary delineated in orange (D). Thresholding and binarization with the Phansalkar method in the peripapillary are shown (E). The large vessel masks in RPC (F) was removed from RPC images to obtain the microcapillary vessel density.

We used the built-in commercial software (Angiovue Analytics version 2016.1.0.26) to obtain the vessel density (VD) of the peripapillary RPC and parafoveal SCP, DCP, and whole retina as well as the FAZ area of the whole retina and the peripapillary structural RNFL thickness. In the RPC layer, we obtained both global data including the entire peripapillary area and data from only the superior quadrant (Fig 2D). The software automatically divided the peripapillary area into four quadrants: superior, inferior, temporal, nasal. For all data reported, the peripapillary region is defined as the donut area between two rings centered on the optic nerve, with inner and outer ring diameters of 2.0 and 4.0mms, respectively (Fig 2D). Centered on the fovea and automatically defined by the machine, the parafoveal area is between rings of inner and outer diameters of 1.0 and 3.0mms, respectively (Fig 2A).

Two independent graders, masked to the diagnosis, analyzed the adjusted flow index (AFI) of the parafoveal SCP, VD of the peripapillary SVC, the vessel length density (VLD) of the peripapillary RPC and parafoveal SCP, and the microcapillary VD of the peripapillary RCP as outlined below.

#### Vessel density

Vessel density was defined as the percentage of the peripapillary region occupied by blood vessels. To obtain VD for the peripapillary SVC, which includes both the RNFL and GCL, images were binarized using the Phansalkar method on ImageJ (developed by Wayne Rasband, National Institutes of Health, Bethesda, MD; available at http://rsb.info.nih.gov/ij/index.html) that applied an 15x15 pixel sampling window for auto local threshold as shown in Fig 2E [34–36]. The binarization threshold for each scan defined the vessels (above threshold) and noise or non-perfusion area (below threshold). The global vessel density was calculated by dividing the area occupied by the blood vessels by the total area of the peripapillary region.

#### Microcapillary vessel density

We analyzed the microcapillary VD of the RPC which was defined as the percentage of the peripapillary region occupied by smaller capillaries, after removing the larger arterioles and venules that are included in the machine calculation of RPC VD. In accordance with previously described methods, we used ImageJ to contrast stretch each image [37], applied the Shanbhag auto-threshold, and then removed perfused peripapillary capillaries of <25 μm^2^ from the image [20]. The remaining major vasculature on the angiogram was used as a mask to remove the large vessels (Fig 2F). The microcapillary density was calculated, similar to the VD, after subtracting the pixels occupied by the large vessels from the RPC layer.

#### Vessel length density (VLD)

VLD represents the ratio of the total length occupied by all the blood vessels in a given area to the total area. The values obtained in VLD are based on the length and number of vessels but not the caliber of vessels. Thus, VLD eliminates the disproportionate representation of large caliber arterioles and venules that is present in the VD parameter. We applied the Phansalkar method to the disc images and used the FAZ background signal as the threshold for macular images according to previously described protocols [38, 39]. All vessels were then skeletonized into 1-pixel wide vessels using skeletonize2D/3D, an open source plugin created for ImageJ (developed by Ignacio Arganda-Carreras; available at http://imagej.net/Skeletonize3D) [40].

#### Adjusted flow index (AFI)

Capillary perfusion is dependent not only on VD, which is based on the caliber, length, and number of vessels, but also on the flow velocity of blood within those vessels. Therefore, we also calculated the AFI, a surrogate measure of blood flow velocity in OCTA. AFI is defined as the average decorrelation value of all vessels in a given area [38]. In ImageJ, this translates to the average decorrelation value of all pixels above the noise threshold defined by the FAZ in the *en face* angiogram.

### Statistical analysis

Statistical analysis was performed using SPSS24 (IBM Corp., Armonk, NY, USA). For each participant, the right eye was selected for analysis except in cases where the right eye failed to meet image quality eligibility or contained retinal pathology, the left eye was used instead. A p value of <0.05 was considered significant for all analysis. We used Independent Sample Student’s T-Test for parametric and Mann-Whitney U for nonparametric distributions to compare VD, VLD, AFI, and FAZ in the macula and or disc as well as structural RNFL thickness of the aMCI/eAD versus control group. To investigate correlations, Pearson correlation was use for parametric and Spearman for nonparametric distributions. We calculated effect sizes with Hedges’ g coefficient.

The reliability and repeatability of the RTvue OCTA system has been previously established in literature [37, 41]. All calculations including the VD, microcapillary VD, VLD, and AFI were graded by 2 independent masked graders (Y.S.Z., N.Z.) and an absolute agreement intraclass correlation (ICC) was calculated using SPSS. In addition, we calculated ICC for consistency between manual RPC VDs obtained by the Phansalkar method and machine RPC VDs.

## Results

The two graders achieved an absolute agreement with an ICC >0.9 (ranging from 0.928–0.987) for all parameters obtained by manual analysis. In addition, the ICC (0.958) showed excellent consistency between the Phansalkar method and automated RPC VD.

The demographic and clinical characteristics of the study participants are shown in Table 1. We recruited 17 aMCI, 4 eAD, and 18 control participants. A total of seven participants were excluded: three aMCI and one control participant for poor image quality, one aMCI for diagnosis of non-amnestic MCI, and one eAD and one control participant for retinal pathology. In the final analysis, we included 13 participants with aMCI, three participants with eAD, and 16 controls. Of those include in the analysis, 16 people in each cohort were included in the macula image analysis and 15 were included in the disc image analysis due to imaging quality inclusion requirements. Subjects were matched 1:1 for age, gender, and race, and thus these characteristics are not statically different between the two groups. There were no significant differences in education, interval between cognitive testing and retinal imaging, or IOP between the two groups. All participants had a best corrected visual acuity of ≥20/40 and IOP ≤21mmHg. As expected, aMCI/eAD participants as a cohort performed significantly worse on all cognitive and memory tests with an average MoCA of 20.25 (equivalent of MMSE of 26 [25]) and had lower global functioning CDR scores.

Results of the macular and disc inner retinal OCTA parameter comparisons between the two groups are summarized in Table 2. Notably, there was a significant decrease (P<0.05) in the parafoveal VD and AFI in the SCP layer of the cognitively impaired group, with a moderate to large effect size (hedges g = 0.81 and 0.70, respectively). No statistically significant difference was found in the mean parafoveal VLDs between the two groups. Additional analysis highlighted in S1 Table shows a significantly lower parafoveal vessel density in the whole retina but not in the DCP layer and no differences in the FAZ area between the two groups.

**Table 2:** Comparison of macular and disc optical coherence tomography angiography parameter means between participants with cognitive impairment and cognitively normal controls.

		aMCI/eAD (n = 16)	Controls (n = 16)	P value^a^
**Parafoveal SCP**			
**VD (%)**	40.67 ± 5.23 (31.1–48.2)	44.50 ± 4.11 (33.7–52.3)	**0.028**^a^
**VLD (%)**	16.41 ± 3.60 (9.28–22.6)	16.73 ± 2.64 (11.85–20.75)	0.772
**AFI**	0.376 ± 0.041 (0.28–0.48)	0.407 ± 0.037 (0.35–0.46)	**0.047**^a^
**Peripapillary RPC**			
**VD (%)**			
*Global*	46.93 ± 5.04 (31.60–51.20)	49.63 ± 1.85 (47.40–53.30)	0.187
*Superior*	47.42 ± 7.31 (26.20–57.95)	49.18 ± 3.91 (43.80–56.25)	0.624
*Capillary*	34.96 ± 4.78 (20.3–39.6)	37.85 ± 1.58 (35.6–41.7)	0.056
**VLD (%)**	15.23 ± 1.87 (11.4–18.4)	16.07± 1.21 (13.5–17.6)	0.156
**Peripapillary SVC**			
**VD (%)**	43.16 ± 4.88 (33.1–48.9)	45.00 ± 3.58 (37.8–53.0)	0.251
**VLD (%)**	16.72 ± 2.01 (12.7–20.0)	17.30 ± 1.41 (13.3–19.2)	0.486

Data reported as mean ± SD with p values from Student’s T-Test and Mann-Whitney U test. Ranges are reported in parenthesis.

^a^statistically significant at p<0.05

Abbreviations: SCP = superficial capillary plexus; RPC = radial peripapillary capillary; VD = vessel density; VLD = vessel length density; AFI = adjusted flow index; SVC = superficial vascular plexus.

In the RPC and SVC layers of the disc (Table 2), there was no significant difference in the VD, VLD, or microcapillary VD between the two groups. In addition, there was no difference in the structural RNFL thicknesses (S1 Table). Despite the lack of statistical significance, the difference in the RPC VD between the groups had a moderate effect size (hedges g = 0.72). In addition, RPC microcapillary VD, which excluded the large vessels, showed a trend towards vessel loss (p = 0.056) and a hedges g of 0.79.

We also found a significant positive correlation (p<0.05) between cognitive performance (MOCA score) on the one hand and both parafoveal SCP VD and peripapillary RPC VLD (Table 3), suggesting that lower cognitive scores were associated with lower capillary density in the macula and optic nerve. Overall, there was a general trend for positive correlation between MoCA and other OCTA parameters, but these did not reach significance.

**Table 3:** Pearson and Spearman correlation between Montreal Cognitive Assessment scores and optical coherence tomography angiography parameters.

	MoCA	
		R value	P value^a^
**Parafoveal SCP**		
**VD**	0.361	**0.043**^a^
**VLD**	0.087	0.636
**AFI**	0.318	0.076
**Peripapillary RPC**		
**VD**	0.325	0.079
**VLD**	0.463	**0.010**^**a**^
**Peripapillary SVC**		
**VD**	0.251	0.181
**VLD**	0.263	0.159

Data reported as r and p values from Pearson or Spearman correlations.

^a^statistically significant at p<0.05

Abbreviations: SCP = superficial capillary plexus; RPC = radial peripapillary capillary; VD = vessel density; VLD = vessel length density; AFI = adjusted flow index; SVC = superficial vascular plexus

## Discussion

Our study shows parafoveal vessel loss and decreased flow in the inner retinal layers on OCTA, suggesting inner retinal hypoperfusion in the macula of those with aMCI and eAD compared to cognitively normal controls. We focused on the SCP which is responsible for the metabolic demands of the parafoveal GCL [15, 42], where cellular loss is seen in AD both histologically and on OCT [16, 17]. Furthermore, while our supplementary analysis demonstrated a lower whole retina VD in the impaired group, we did not see a difference in the DCP layer which again supports the inner retina as the target of pathology. Our data expands upon previous evidence of cerebral and retinal vessel loss in AD. Bell et al. [43] have shown histological loss of small cerebral blood vessels. In the eye, Bulut et al. [21]have shown decreased macular inner retinal vessels on OCTA in a cohort with more advanced AD. However, this finding was not replicated in MCI subjects by Jiang et al. [20], who only showed capillary alterations on OCTA in the deeper retinal layers perhaps due to not specifically selecting for an aMCI population. Our data indicate that vessel dropout in the inner retina may occur as early as in the eAD and aMCI stage. This loss of vasculature has been postulated to be precipitated by impairment of Aβ clearance, the constrictive and anti-angiogenetic effects of Aβ, and amyloid angiopathy [44–47].

In addition to capillary loss, we saw decreased AFI in the parafoveal SCP suggesting that hypoperfusion may be associated with decreased flow in the remaining vessels. While prior studies using retinal function imager and laser flowmetry in MCI showed flow reduction in larger vasculature such as the parafoveal arterioles and venules [12] and the temporal retinal vein [13], our results confirm that these results are also reflected in the capillary circulation as seen on OCTA. However, the lack of significant decrease in pixel-wide SCP vessel density, as measured by the VLD, in the face of a significantly decreased overall SCP VD could suggest that macular vessel loss disproportionately affects the larger vessels. Our finding emphasizes the importance of considering blood vessel size in OCTA studies.

In our cognitively impaired cohort, we did not find significant differences in the FAZ area, unlike a previous study by O’bryhim et al. [22] who reported larger FAZ areas in their pre-AD subjects. There are many potential confounders reported to affect the FAZ area, including age, race, gender, and axial length [48–50]. In fact, axial length alone accounted for errors that ranged from -20% to +51% in the FAZ area in one report [50]. In addition, FAZ parameters are associated with a wide range of variability even in healthy individuals [2, 48], making the FAZ more robust for studying longitudinal changes in the same individual than comparing groups of subjects, especially when the groups are not matched for all the potential confounders [48]. In fact, one of the main differences between our study design and the O’bryhim study is that we age-, gender- and race-matched our study populations to avoid many of these potential confounders, which could partly explain the discrepancy. Therefore, we believe the controversy regarding the role of the FAZ can only be resolved by large population studies that adjust for age, gender, race, and axial length.

We did not find a significant difference between the two cohorts in any of the peripapillary parameters, including global or focal superior quadrant vessel density. Based on histological [18] and structural OCT imaging [17, 51] studies that show thinning of the peripapillary RNFL especially in the superior quadrant of those with AD, we expected to see peripapillary RPC vascular affection [42]. However, we found that the microcapillary VD of the RPC layer, which is the density of only the small vessels, was lower in the cognitively impaired group compared to controls with a trend towards statistical significance. Furthermore, we found a significant positive correlation between pixel-wide peripapillary capillary density (VLD) and MoCA scores but not between the overall peripapillary VD and MoCA scores in the entire study cohort. This emphasizes the importance of small capillary changes in the disc as the pixel-wide vessel density (VLD) removes the disproportionate influence of larger vessels on density calculations. Future OCTA studies of the peripapillary region in AD or MCI should consider focusing on the smaller capillaries, where pathological vessel loss may be overlooked when using the standard automated VD parameters.

We postulate that the lack of significant findings in the disc compared to findings in the macula could be related to our small sample size or anatomical differences between the two areas. Given that the effect size for RPC VD difference between the groups was moderate (hedges g = 0.72) and the microcapillary VD showed a trend toward significance, the lack of statistical significance may be attributed partly to our small sample size. On the other hand, the RPC contains multilayered capillaries that overlap on *en face* OCTA images, complicating the ability to detect small capillary losses [42, 52]. Another possibility is that the parafoveal SCP originates largely from the retinal circulation, which has been shown to be affected in AD, [53, 54] whereas the peripapillary RPC receives additional perfusion from the choroidal ciliary vessels [42, 55]. These variables may in combination explain why early perfusion defects may be detected in the macula before the disc. Future multi-center studies with larger sample sizes are needed to further examine this question.

Our aMCI/eAD cohort had similar RNFL thickness to our controls. While overwhelming evidence point to RNFL thinning in later stages of AD [17, 51], our finding is not surprising in early disease. Individuals with MCI or early-moderate AD have been shown to either have no difference, [56] decreased, [57] or even increased [58] RNFL thickness when compared to cognitively normal controls. Our group [58] recently found an inverse correlation between cognitive scores and RNFL thicknesses in aMCI. We hypothesized that a transient gliotic change occurs in those with MCI prior to RNFL thinning in AD. Future longitudinal investigations may elucidate the temporal changes of RNFL with disease progression.

Lastly, we found a significant positive correlation between MoCA performance and parafoveal SCP VD as well as peripapillary RPC VLD in the entire cohort of study participants. Bulut et al. [21] previously found similar correlations between MMSE scores and SCP VD in a cohort of AD and cognitively normal participants. In our study with eAD/MCI patients, where cognitive score differences between groups and individuals are much narrower, we were able to also detect correlations between OCTA parameters and MoCA scores likely in part because MoCA is a superior test in distinguishing more subtle changes in cognition [27]. In the brain, Lim et al. [59] found that decreased flow velocity and increased pulsatility in the cerebral arteries as measured by doppler was associated with lower cognitive scores in those with AD at 1 year follow-up. Our data suggest that vessel density in the retinal may also have a potential role in tracking cognitive decline, a hypothesis that needs to be validated in longitudinal studies.

Taken together, our results show parafoveal hypoperfusion in subjects with early cognitive impairment as well as a correlation between cognitive performance and vascular density in the macula and disc. These relationships support the potential for retinal capillary imaging to serve as a biomarker for early cognitive impairment. Future studies with larger samples and longitudinal design will be important to elucidate whether these angiographic biomarkers have sufficient sensitivity and specificity to screen for cognitive impairment, monitor disease progression, or predict future cognitive decline. The clinical impact of these studies would be tremendous, given the often unpredictable clinical course of pre-AD individuals [4] and the invasive and costly nature of current biomarkers that prohibits their widespread implementation [7, 8]. There is also currently a largely unmet need for validated, non-invasive tools that identify subjects with early impairment and classify them according to their risk of progression. These tools would significantly facilitate clinical trials and research studies, allowing early therapeutic interventions to be targeted to the subjects who are most at-risk [7].

The strengths of this study include rigorous neuropsychological testing to identify a strictly defined population of participants in mild stages of cognitive impairment (average MoCA of 20, equivalent to MMSE of 26) with predominant memory domain impairment. We used a tightly matched case-control design in order to eliminate confounding effects of age, gender, and race on vascular parameters and RNFL thickness [60–63]. In addition, we carefully excluded other neurological and retinal conditions by closely examining participants, taking careful histories, and studying their electronic health records. To ensure rigor of the analysis, our two separate graders achieved an absolute ICC of >0.9 on parameters that required manual analysis.

Limitations of this study include the small sample size which can be attributed to our careful selection of aMCI participants, stringent inclusion, exclusion, and image quality criteria. However, the small study size does not account for our parafoveal findings which have moderate to large effect sizes. Our study population is over age 70 and thus, we were careful to use high quality images which could be limited by the presence of cataracts or reduced physical abilities of participants. We did not obtain axial length for correction of magnification error. Lastly, due to the cross-sectional design of our study, we can only show a correlation but cannot establish causation between vascular changes, structural RNFL changes, and cognitive pathology.

## Conclusion

In conclusion, our study shows that compared to matched cognitively normal controls, participants with early cognitive impairment demonstrated significantly decreased superficial parafoveal vessel density and blood flow. In addition, we found that parafoveal and peripapillary densities were positively correlated with the MoCA, a measure of overall cognitive impairment. Most importantly, we demonstrated the role of OCTA in detecting early capillary changes, which may represent potentially early, non-invasive biomarkers of AD. Future directions include a larger cohort as well as longitudinal studies that examine the temporal relationship between vascular damage and pathological loss of ganglion cells, their nerve fibers, and cognitive decline.

## Supporting information

S1 Table Comparison of additional macular and disc optical coherence tomography angiography parameter means between participants with cognitive impairment and cognitively normal controls. Data reported as mean ± SD with p values from Student’s T Test and Mann-Whitney U test. Ranges are reported in parenthesis. ^a^statistically significant at p<0.05 Abbreviations: aMCI = amnesic mild cognitive impairment; eAD = early Alzheimer’s Disease; RNFL = radial nerve fiber layer; FAZ = foveal avascular zone; DCP = deep capillary plexus; VD = vessel density
(DOCX)

S1 Dataset Demographic, cognitive, and optical coherence tomography angiography raw data in participants with cognitive impairment and cognitively normal controls. (XLSX)
