# Supplementary material for: Parafoveal vessel loss and correlation between peripapillary vessel density and cognitive performance in amnestic mild cognitive impairment and early Alzheimer’s Disease on optical coherence tomography angiography
Source: PLoS One. 2019 Apr 2;14(4):e0214685. doi: 10.1371/journal.pone.0214685 (PMC6445433; doi:10.1371/journal.pone.0214685)
Supplement: S1 Table — Data reported as mean ± SD with p values from Student’s T Test and Mann-Whitney U test. Ranges are reported in parenthesis. astatistically significant at p<0.05 Abbreviations: aMCI = amnesic mild cognitive impairment; eAD = early Alzheimer’s Disease; RNFL = radial nerve fiber layer; FAZ = foveal avascular zone; DCP = deep capillary plexus; VD = vessel density (DOCX) [file pone.0214685.s001.docx]

**S1 Table.** Comparison of additional macular and disc optical coherence tomography angiography parameter means between participants with cognitive impairment and cognitively normal controls

|  |  | | **aMCI** (n=13)/ **eAD** (n=3) | **Controls** (n=16) | **P value^a^** |
| --- | --- | --- | --- | --- | --- |
| **RNFL** |  | |  |  |  |
|  | **Thickness (µm)** | |  |  |  |
|  | *Global* | | 97.73 ± 11.42  (82 - 121) | 99.40 ± 11.83  (75 - 120) | 0.698 |
|  | *Superior* | | 110.67 ± 13.72  (89.5 - 137) | 111.47 ± 17.21  (77.5 - 139) | 0.889 |
| **FAZ Whole Retina** | | |  |  |  |
|  | | **Area (mm^2^)** | 0.247 ± 0.092  (0.106-0.43) | 0.271 ± 0.135  (0.055-0.434) | 0.550 |
| **Parafoveal DCP** | | |  |  |  |
|  | **VD (%)** | | 49.51 ± 4.66  (41.3 – 59.1) | 50.05 ± 2.75  (45.5 – 56.0) | 0.691 |
| **Parafoveal Whole Retina** | | |  |  |  |
|  | **VD (%)** | | 48.73 ± 6.92  (32.9 – 60.4) | 54.90 ± 4.47  (42.2 - 62.4) | **0.004^a^** |

Data reported as mean ± SD with p values from Student’s T Test and Mann-Whitney U test. Ranges are reported in parenthesis.

^a^statistically significant at p<0.05

Abbreviations: aMCI= amnesic mild cognitive impairment; eAD= early Alzheimer’s Disease; RNFL= radial nerve fiber layer; FAZ= foveal avascular zone; DCP= deep capillary plexus; VD= vessel density
